# Huge Bullous Keratopathy following Trauma

**Published:** 2010-10

**Authors:** Mahmoudreza Panahibazaz, Mitra Zamani, Farinaz Borna

**Affiliations:** Imam Khomeini Hospital, Jundishapur Medical University, Ahvaz, Iran

A 20 year-old man presented with redness, severe visual loss and progressive protrusion of a horn-like mass from the palpebral fissure of his left eye (Fig. 1). He reported gradual appearance of a bubbly tissue on the left cornea progressively growing out of the palpebral fissure over the past several months.

He had history of ocular trauma with a piece of glass from an exploding vial with unknown contents which had been thrown into a fire 8 years before, at the age of 12 years. Ocular examination 5 days after trauma at an emergency ophthalmic facility had revealed visual acuity of 20/30 and a 3 mm self-sealed full-thickness corneal laceration in the left eye and a glass-like intraocular foreign body in the anterior chamber. Topical betamethasone drops had been prescribed and an orbital CT-scan was requested.

The patient was not returned by his parents to the clinic until 7 months later. By that time, visual acuity had deteriorated to counting fingers at 50 cm, and the eye developed 3+ to 4+ corneal edema with inferior limbal vascularization. Timolol 0.5% eye drops and a sodium chloride 5% ointment were prescribed and the patient was considered for penetrating keratoplasty, which was refused by his parents for 8 years.

When the patient reached 20 years of age, he independently decided to receive a corneal graft. Upon presentation, visual acuity had further decreased to hand motions, however red-green color perception was good and relative afferent pupillary defect was negative. The left cornea was edematous and opaque with inferior peripheral vascularization and a huge epithelial bulla resembling a crystalline horn protruding at least 3 mm out of the palpebral fissure (Fig. 1). B-scan ultrasonography revealed an attached retina and CT-scan ruled out the presence of an intraocular foreign body preoperatively.

At the time of penetrating keratoplasty, the tip of the bulla was ruptured with the cross-hair guide of the Barron corneal suction trephine (Katena Products Inc., Denville, NJ, USA) and the bulla collapsed. After excising the recipient cornea, the iris, pupil, and crystalline lens were normal and there was no sign of foreign bodies; it was therefore presumed that the glass-like shadow reported in the anterior chamber on initial examination consisted of particles of unknown composition contained within the vial which had gradually been absorbed. Figure 2 shows the postoperative appearance of the eye 6 weeks after penetrating keratoplasty.

## DISCUSSION

The lattice-like arrangement of collagen fibrils in the corneal stroma accompanied by barrier and pump functions of the corneal endothelium are the most important factors for maintaining corneal transparency, deturgescence and steady-state thickness.^1^

Endothelial cells have macula occludens-type junctions at the apical portion of their lateral membranes which prevent massive transport of fluid from the anterior chamber into the corneal stroma. Na^+^/K^+^-ATPase pumps are located along these tight junctions.^2^ The barrier function of the endothelium depends on the level of calcium, glutathione and adenosine in the aqueous humor. Elevated calcium concentration and reduced glutathione or adenosine levels can disrupt this barrier function resulting in corneal edema^1^.

The effects of drugs and preservatives on endothelial cells and barriers depend on the nature of the substances and their concentration.^2^ Some substances can cause irreversible damage to endothelial cells even in very low concentrations during the immediate post-exposure period.^2^

In our patient, progressive corneal edema and eventually corneal decompensation was observed 8 months after penetrating trauma and exposure to an unknown substance. Histopathologic examination of the excised corneal button revealed severe endothelial cell destruction and reduction in density to less than the minimum level required to maintain corneal deturgescence, i.e. 515 to 549 cells/mm^2^.^2^

Chronic corneal edema in the patient described herein, led to formation of a huge horn-like epithelial bulla protruding at least 3 mm out of the palpebral fissure with an unusual appearance. A review of literature failed to disclose similar reports on bullous keratopathy with such configuration.

## Figures and Tables

**Figure 1:**
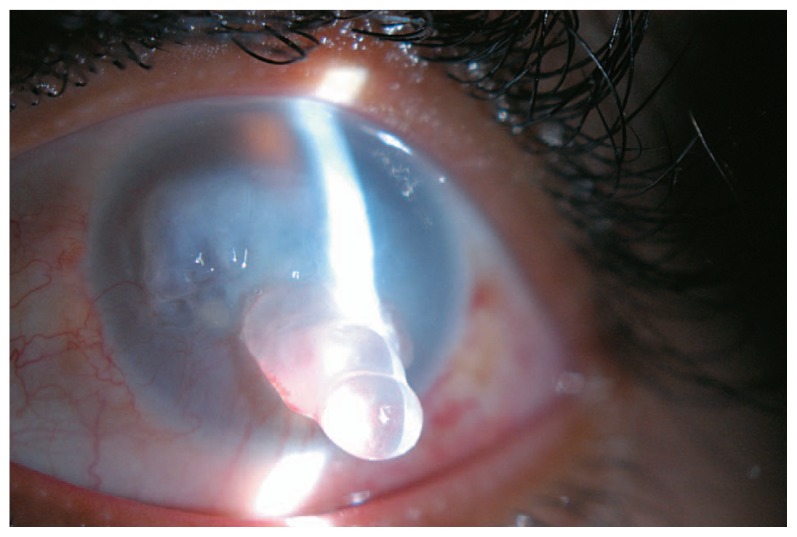
Left eye of the patient, note the edematous and opaque cornea and the large horn-like epithelial bulla.

**Figure 2:**
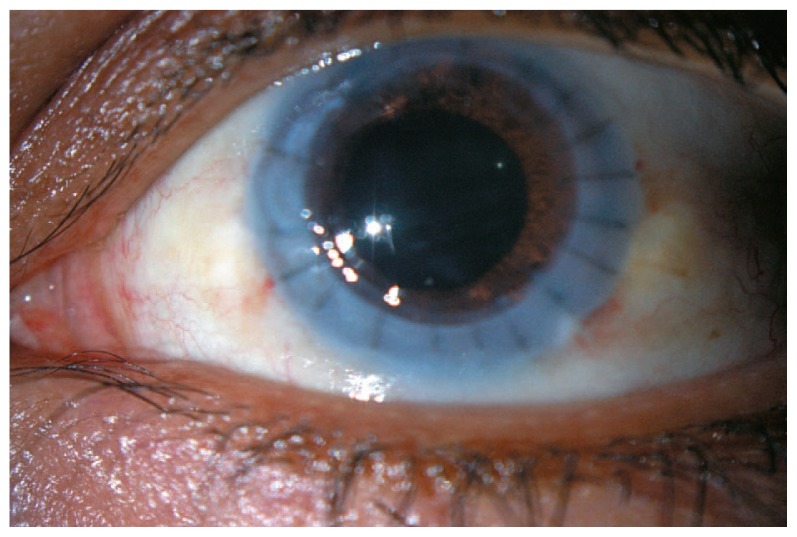
Left eye of the same eye after penetrating keratoplasty.
